# The Value of Patient-administered Depression Rating Scale in Detecting Cognitive Deficits in Depressed Patients

**DOI:** 10.4021/jocmr2010.02.224w

**Published:** 2010-02-05

**Authors:** Charles Lung-Cheng Huang

**Affiliations:** a Department of Psychiatry, National Taiwan University Hospital Yun-Lin Branch; b Institute of Medicine, Kaohsiung Medical University.

## Abstract

**Background:**

The aims of this study was to clarify how accurate the depressed patients perceive their cognitive symptoms, and verify the appropriateness of the depressive rating scales in evaluating cognitive deficits in major depressive disorder (MDD) patients.

**Methods:**

The subjects consisted of 19 well-characterized medication-free patients with MDD and 19 healthy volunteers. The clinical and neuropsychological assessments, including Hamilton Rating Scale for Depression (HAM-D), Taiwanese Depression Questionnaire (TDQ), Finger Tapping Test, Wechsler Memory Scale-Revised, Stroop Color-Word Test, and Continuous Performance Test, were administered at the time of recruitment and repeated six months after treatment.

**Results:**

Depressed patients exhibited significant impairment in several neurocognitive domains. Neurocognitive impairment was correlated with both the affective and somatic factors, but not with the cognitive factors of TDQ. The similar results were shown after 6-month treatment.

**Conclusions:**

Our results suggest that the MDD patients do not perceive their cognitive dysfunction correctly. The depression rating scales, especially the patient-administered scale, need to be validated for measuring neurocognitive deficits of MDD patients in the future, if cognitive component is suspected as one of the major domains of self-rating scale.

**Keywords:**

Major depressive disorder; Cognitive deficits; Depression rating scale

## Introduction

There is growing evidence of significant impairment in neurocognitive functions in major depressive disorder [1]. Depression-related disturbances of cognitive function have been demonstrated in a range of domains, including attention [2-4], memory [5,6], executive functions [7,8], and psychomotor functions [9]. However, findings to date are varied [10] with many factors potentially contributing to inconsistencies between studies, including age, hospitalization, severity and subtype of depression, and most importantly, the effect of psychotropic medication [11].

Although cognitive deficits are a common and potentially debilitating feature of major depressive disorder (MDD), such cognitive impairments are seldom measured with objective methods as a clinical routine. There are only limited items that pertain to neurocognitive impairments in the most widely used depression rating scales, for example, the Hamilton Depression Rating Scale [12] and the Beck Depression Inventory [13]. Although a recently developed depression questionnaire tried to incorporate more comprehensive cognitive items [14,15], the validity concerning the neurocognitive domain of depression has not yet been confirmed.

By testing a well-characterized group of medication-free patients with non-psychotic, unipolar major depressive episodes on a comprehensive neurocognitive test battery; this study aims to explore the correlation between the neurocognitive deficits and the depressive symptoms. By shedding some light on how accurate the depressed patients perceive their cognitive symptoms, we may be able to verify the appropriateness of the depression rating scales in evaluating cognitive deficits in MDD patients.

## Patients and Methods

### Subjects and design

Patients with non-psychotic MDD were recruited from the outpatient clinic of a general hospital. The entry criteria included: (1) aged 18 - 65 years and able to communicate with speech or writing; (2) fulfilling DSM-IV criteria for a current major depressive episode and free of previous manic/hypomanic/mixed episodes; (3) entirely psychotropic medication-free currently and for at least six weeks before recruitment; (4) having scored at least 16 on the Hamilton Depression Rating Scale (17-item); (5) except for MDD, free of any concurrent or past axis I or axis II psychiatric disorder, including dementia; (6) free of neurological diseases, illicit substance abuse or dependence as determine by clinical history and examination.

A total 19 healthy volunteers were recruited from the community by advertisement. These volunteers were interviewed by a senior psychiatrist using the Chinese version of the Mini International Neuropsychiatric Interview [16] to exclude individuals with mental illnesses.

All subjects signed informed consent statement. The study protocol was approved by local Ethical Committee for Human Research.

The demographic data and medical information of subjects were collected, and the clinical and neuropsychological assessments were administered at the time of recruitment and six months after treatment. The instrument-based clinical and neuropsychological assessments are as follow:

### Clinical assessments

1. Hamilton Rating Scale for Depression (HAM-D): The 17-item HAM-D is a clinician-rated scale. It is the most widely used depression scale in both clinical and research settings [12].
2. Taiwanese Depression Questionnaire (TDQ): The 18-item TDQ is a patient-administered measure of depression. It is a culturally relevant questionnaire, which is adaptable for screening patients with depression in local communities. The TDQ is designed to collect data representative of the past seven days, and the cut-off score is 19 [14].

### Neuropsychological assessments

1. Finger Tapping Test (FTT): FTT measures the tapping speed of the index finger of each hand [17].
2. Wechsler Memory Scale-Revised (WMS-R): The WMS-R is a substantial revision of the WMS. The subtest raw scores are combined and the following indexed are computed: (1) Verbal Memory Index, (2) Visual Memory Index, (3) General Memory Index, (4) Attention/Concentration Index, and (5) Delayed Recall Index [18].
3. Stroop Color-Word Test (SCWT): A test of executive functioning, the SCWT requires the patient to inhibit a dominant set to read a word in favor of a nondominant set to reported the color of ink in which a word is printed [19].
4. Continuous Performance Test (CPT): The CPT is a test of attention. This test requires the patient to rapidly respond to sequentially presented stimuli. It assesses (1) sustained attention; and (2) reaction time [20-21].

### Data analyses

Rates and proportion with a two-tailed 95% CI were calculated for the variables of interest. Wilcoxon matched-pairs signed ranks test was used to compare the ages, HAM-D scores, and variables of cognitive measurement between patients and nonpatients. Cross table Chi-square was used to compare the sex, education level, and marital status between both groups. Factor analysis of the TDQ was performed to yield three solutions: the cognitive, affective, and somatic factors (Table 1). The principal components of HAM-D, namely the insomnia/hypochondriasis, anxiety, and core depression factors, were adapted from the study of Samuels et al [22]. By using Pearson’s product moment correlation coefficient, the correlation between cognitive functions and principal components of HAM-D and TDQ was tested with partial correlations controlling for age. Furthermore, the correlation of difference in neurocognitive performance and difference in TDQ scores after the six-month treatment was also tested. All data were analyzed using the SPSS for Windows package, Release 10.0 [23].

**Table 1:** The factor Structure and Lodgings and Factors Cronbach’s α for the TDQ

	Factor 1	Factor 2	Factor 3
	Cognitive	Affective	Somatic
**Factor 1 : Cognitive**			
3. I felt more agitated than before	**.46**	.07	-0.09
9. I felt upset	**.64**	-.18	.19
10. I had poor memory	**.63**	-.02	.04
11. I could not concentrate when doing things	**.50**	-.11	.01
12. I was slower in thinking and doing things than before	**.68**	.04	-.22
13. I felt less confident than before	**.74**	.16	-.31
14. I tended to look at the dark side of everything	**.64**	.10	-.19
15. I felt miserable and even want to die	**.43**	-.02	.18
18. I felt worthless	**.65**	-.38	.63

**Cronbach’s α**	**.86**		

**Factor 2: Affective**			
1. I often felt like crying	-.14	**.40**	-.18
2. I felt blue and depressed	.24	**.89**	-.52
5. I had poor appetite	.05	**.50**	.37
7. I felt uneasy, uncomfortable	-.13	**.64**	.32
8. I felt tired and weak (“Xu”, “mo wan qi”)	.23	**.68**	-.11
16. I lost interest in everything	.32	**.48**	.13
17. I felt sick. (Headache, dizziness, palpitation, or abdominal distress)	.00	**.59**	.39

**Cronbach’s α**		**.95**	

**Factor 3: somatic**			
4. I had trouble in sleeping...	-.12	.15	**.72**
6. I frequently had chest tightness(“sim-guan-tau-bang-bang”)	-.25	.36	**.63**

**Cronbach’s α**			**.87**

## Results

### Neuropsychological measures for patients and controls group

The subjects consisted of 19 patients and 19 controls. In the patients group, there were 14 subjects with first episode, and 5 with relapse or recurrence. The mean episode length was 13.6 ± 16.1 (mean ± SD) weeks. There was no significant difference in age, gender, educational level, or marital status between the patient and controls groups (Table 2).

**Table 2:** Demographic Data and Neuropsychological Measures for Patients and Controls

	Major Depression		Normal Controls		Z	Sig.
	Mean ± SD	N	Mean ± SD	N		
**Sex**						
Male		5		10		.09
Female		14		9		
**Educational level**						
Below junior high school		4		0		.11
Above junior high school		15		19		
**Marital status**						
Married		8		12		.33
Single		11		7		
**Age**	33.16 ± 9.77	19	36.05 ± 7.91	19	-.86	.40
**HRSD-17**	21.47 ± 5.00	19	0.33 ± 0.76	19	-5.35	**
**WMS-R**						
Verbal MEM.I	80.08 ± 18.06	19	93.25 ± 10.76	19	-2.38	*
Visual MEM.I	87.58 ± 24.13	19	115.25 ± 13.95	19	-2.71	**
General MEM.I	84.42 ± 22.84	19	100.00 ± 9.38	19	-2.43	*
Attention/Concentration	102.42 ± 8.71	19	101.14 ± 10.25	19	-0.77	.45
Delayed Recall I	90.07 ± 19.13	19	108.07 ± 11.99	19	-2.86	**
**FTT**						
Dominant hand	35.63 ± 14.86	19	61.46 ± 10.63	19	-3.81	**
**SCWT**						
Test time	118.8 ± 76.87	19	70.85 ± 20.27	19	-2.42	*
**CPT**						
d’ MASKED	3.83 ± .91	19	3.70 ± .91	19	-.41	.71

^*^p < 0.05 (two-tailed); ^**^p < 0.01 (two-tailed). ^a^HAM-D-17: 17-item Hamilton Rating Scale for Depression; WMS-R: Wechsler Memory Scale–Revised; CPT: Continuous Performance Test; FTT: Finger Tapping Test, SCWT: Stroop color-word test. ^b^Mann-Whitney U test was used to compare the ages and variables of cognitive measurement in both groups. Cross table chi-square was used to compare the sex, education level, and marital status. ^c^Significance at the study-wise type I error rate of 0.05 (2-tailed) after adjustment. P value with Hochberg’s Sharpened Bonferroni correction.

The results indicated significant differences between depressed patients and nondepressed healthy controls on WMS-R (Verbal Memory Index, Visual Memory Index, General Memory Index, and Delayed Recall Index), SCWT, and FTT (Table 2). There were no significant differences between the two groups in CPT, or Attention/Concentration Index of WMS-R.

### Relationships between cognitive deficits and depressive symptoms

Factor analyses of the HAM-D and TDQ yielded three solutions, respectively. The cognitive deficit as measured in WMSR was correlated with the insomnia/hypochondriasis factor of HAM-D; while deficit in FTT was correlated with both the anxiety and core depression factors (Table 3). The cognitive deficits shown in WMSR Visual Memory Index, and SCWT were correlated with somatic factor of TDQ; while WMSR Verbal Memory Index and General Memory Index were correlated with the affective factor (Table 4). No neurocognitive impairment was correlated with the cognitive factor of either HAM-D or TDQ.

**Table 3:** Neurocognitive Performance versus HRSD Scores and Principal Components, Partial Correlation Controlling for Age

	Sum	Factor 1	Factor 2	Factor 3
	(r)	(r)	(r)	(r)
**WMSR**				
Verbal MEM.I	-.50	-.40	-.06	-.30
Visual MEM.I	.17	-.07	-.10	-.44
General MEM.I	-.49	-.62^*^	.00	-.41
Attention/Concentration I	.37	-.01	-.10	.07
Delayed Recall I	-.28	-.13	-.01	-.50
**FTT**				
Dominant hand	.42	.05	.55	-.57^*^
**SCWT**				
Test time	.14	-.32	.06	-.15
**CPT**				
d’ MASK	-.52	-.51	.02	-.46

^*^p < 0.05 (two-tailed). ^a^WMS-R: Wechsler Memory Scale–Revised; CPT: Continuous Performance Test; FTT: Finger Tapping Test, SCWT: Stroop color-word test. ^b^Factor 1: insomnia/hypochondriasis factor; Factor 2: anxiety factor; Factor 3: core depression factor.

**Table 4:** Neurocognitive Performance versus TDQ Scores and Principal Components, Partial Correlation Controlling for Age

	Sum	Cognitive Factor	Affective Factor	Somatic Factor
	(r)	(r)	(r)	(r)
**WMSR**				
Verbal MEM.I	-.50	-.31	-.78^**^	.33
Visual MEM.I	.17	.06	-.07	.68^*^
General MEM.I	-.49	-.39	-.63^*^	.34
Attention/Concentration I	.37	.59	-.09	.54
Delayed Recall I	-.28	-.08	-.53	.43
**FTT**				
Dominant hand	.42	.22	-.08	.63
**SCWT**				
Test time	.14	-.11	-.46	.92^***^
**CPT**				
d’ MASK	-.52	-.61	-.58	-.19

^*^p < 0.05 (two-tailed); ^**^p < 0.01 (two-tailed); ^***^p < 0.001 (two-tailed). aWMS-R: Wechsler Memory Scale–Revised; CPT: Continuous Performance Test; FTT: Finger Tapping Test, SCWT: Stroop color-word test.

The correlation between difference in neurocognitive performance and difference in TDQ scores and principal components after the six-month treatment are showed in Table 5. The changes of FTT and WMSR Attention/Concentration Index were correlated with the change in TDQ affective factor. The changes in FTT, SCWT, and WMSR Visual Memory Index, General Memory Index, and Delayed Recall Index were correlated with the change in TDQ somatic factor. The changes in CPT and WMSR General Memory Index change were correlated with the change in the total score of TDQ. The change in neurocognitive functions was not correlated with the change in the cognitive factor of TDQ after the six-month treatment.

**Table 5:** Change in Neurocognitive Performance versus Change in TDQ Scores and Principal Components, Partial Correlation Controlling for Age

	Sum	Cognitive Factor	Affective Factor	Somatic Factor
	(r)	(r)	(r)	(r)
**WMSR**				
Verbal MEM.I	.84	.64	-.11	.91
Visual MEM.I	-.66	-.80	-0.11	1.00^***^
General MEM.I	1.00^**^	-.01	-.89	1.00^***^
Attention/Concentration I	-.13	.28	-1.00***	.63
Delayed Recall I	.64	.43	-.50	1.00^***^
**FTT**				
Dominant hand	.29	-.19	1.00^***^	1.00^***^
**SCWT**				
Test time	.29	.19	-.40	1.00^***^
**CPT**				
d’ MASK	-1.00^**^	-.61	-.58	-.19

^*^p < 0.05 (two-tailed); ^**^p < 0.01 (two-tailed); ^***^p < 0.001 (two-tailed). aWMS-R: Wechsler Memory Scale-Revised; FTT: Finger Tapping Test, SCWT: Stroop color-word test.

## Discussion

### Neurocognitive impairment in MDD patients

The present study demonstrates significant neurocognitive deficits in drug-free adult outpatients with non-psychotic depression, compared with healthy controls. The neurocognitive deficits in MDD patients were found in multiple domains, including psychomotor functioning, memory, and executive functions. These findings are similar to those of previous studies [5,24,25], while previous studies reported that these abnormalities appear particularly pronounced in elderly patients and among midlife patients with melancholic or psychotic characteristics, our findings indicated widespread neurocognitive impairments in younger adult patients (average age was 33.2 years) with non-psychotic MDD. Since our patients were medication free, such trend cannot be attributed to the confounding effects of psychotropic medication.

### Do depressed patients perceive their cognitive symptoms correctly?

Our data shows that the patients with depression have a wide range of neurocognitive impairments compared to normal subjects. The neurocognitive deficits in depression are correlated with the affective or somatic components, but not the cognitive component, of depression rating scales. Furthermore, the changes in neuropsychological measures are correlated with the changes in affective and somatic components, but not the cognitive component of TDQ. In other words, the subjective cognitive symptoms are not correlated with the objective measures of neurocognitive impairments. Currently, few studies have examined the validity of cognitive symptoms in depressive patients. Even in the context of suspected cognitive disorder, the validity of memory complaints is subject to debate [26,27]. In cross-sectional studies in elderly adults, memory complaints were associated with depressive symptoms [28,29], but not with their levels of cognitive functions or memory performance [26,27]. In contrast, Antikainen et al [30] reported that patients complaining of memory disturbances had higher BDI and HDRS scores than patients not complaining, and that they also did less well in objective memory performances but not in other cognitive functions. However, their sample was characterized by chronic inpatients under mediation treatment, which was different from ours.

Taken together, our results suggest that the depressive patients do not perceive their cognitive dysfunction correctly. The depression rating scales, especially the patient-administered scale, need to be validated in neurocognitive domains for MDD patients in the future, if cognitive component is suspected as one of the major domains of self-rating scale.

Generalization of our results must be viewed with caution considering the outpatient sample, the relatively small sample size, increased α-error due to multiple comparison, and possible confounding medication effect during follow-up.

### Conclusions

Pronounced neurocognitive impairment was found in our sample of adult outpatients with non-psychotic MDD. Since this result not attributable to the confounding effects of psychotropic medication, it could therefore serve as an objective marker of brain dysfunction in depression. The observation that cognitive impairments in depressed patients might correlate with affective or somatic factors, but not with cognitive factor, indicating the tendency of inaccurate report of cognitive symptoms in depressed patients. Since the validity of the cognitive component of patient-administered depression rating scale is poor, clinicians may need more objective neuropsychological tests to determine the degree of neurocognitive impairments in depressed patients.
